# Academy of Stomatology

**Published:** 1904-10

**Authors:** 

**Affiliations:** Academy of Stomatology


### ACADEMY OF STOMATOLOGY.

A REGULAR meeting of the Academy of Stomatology of Philadelphia was held at its room, 1731 Chestnut Street, on the evening of April 26, 1904, the President, Dr. L. Foster Jack, in the chair. A paper, entitled "The Superiority of High-Fusing Porcelain for restoring Teeth," was read by Dr. Herbert Locke Wheeler, of New York City.

(For Dr. Wheeler's paper, see page 414.)

*Dr. Gardiner.*—I have listened with a great deal of interest to the paper, and while I have been using porcelain to a considerable extent for a number of years, I have made no comparative tests, and I do not feel that I am qualified to discuss the paper along the lines the essayist has chosen.

*Dr. E. C. Kirk.*—This paper seems important in that it represents a stage in the development of our ideas about the use of porcelain which we must necessarily take up and go through before we really know anything at all about the subject of porcelain, which seems quite analagous to that of amalgam. There we have a composite material. Dentists have been using it since 1835 or 1840, and we have been placing amalgam in teeth in an empirical, haphazard sort of way, assuming that amalgam was a uniform thing and that any combination of metals with mercury made an amalgam which for our purposes was the same as any other amalgam. After awhile we experienced many failures as the result of the fact that it was almost as variable in composition, considering the way in which it was manipulated, as the nondescript material served in boarding-houses under the name of "hash." Then we did what should have been done long before, and that was to investigate this material in order that we might obtain a definite knowledge of its properties as data to work upon. So, the profession has taken up enthusiastically the question of porcelain, because the material has qualities that recommend it and offer relief from two things, of which we should have been relieved long ago,—viz., the drudgery of hammering gold into teeth, and the barbaric display of gold in the human denture. We took it up heartily, because of its artistic quality and for relief from amalgam and gold.

We have had dealt out to us a variety of things called porcelain, and the essayist has truthfully directed our attention to the fact that all these things supplied as porcelain are by no means porcelain. We have no right to classify them under such a general term as that.

It is true that the distinction between high- and low-fusing bodies, based upon the melting-point of gold, is an arbitrary distinction. As the essayist says, it is difficult or almost impossible to say where glass begins and porcelain ends, as it is difficult to say where iron leaves off and steel begins. It is true that the tendency of so-called low-fusing porcelains, which are nothing more than modified glasses, to disintegrate in the fluids of the mouth is due to their basic element, potash or soda, on which depends their low-fusing quality. I do not know much about porcelain and glass in relation to inlay work, but there are some data in relation to glass and porcelain which have a distinct bearing on the case. It is not generally known to those who have not investigated the subject that glass will disintegrate even under the action of distilled water, and in contact with certain corrosive fluids, strong alkaline solutions, and strong or even dilute acids; so that in the storing of chemicals and in the production of chemical glassware it is of the utmost importance, particularly in photographic work, to have a special composition of glass that will not disintegrate under this influence and contaminate the contents of the vessels, spoiling them as chemical reagents. The disintegration of glass under these circumstances is well known in chemical laboratories. If this be true of glassware, it is also true of these low-fusing porcelains.

Another item of importance is in regard to the use of felspar and the disintegration of this felsparic rock we speak of as a name for a class of minerals as complex as the alums. The tendency of this material to disintegrate is in proportion to its alkalinity. That is an important question in relation to the manufacture of porcelain. It is undoubtedly true that the composition of this material is one which greatly affects its durability; and it is equally true that the higher the fusing-point, the less tendency is there to disintegrate. We are only finding this out in dentistry by experience. We should have known that in advance from the study of other related facts. It is also true that the question of preservation of color in the inlay under the high temperature of the furnace depends largely upon the composition, and also upon the fusing-point. The more fluid the porcelain glass becomes—the so-called porcelain glass—the more tendency there is for the oxides which form the color ingredient to undergo chemical changes that cause their disappearance.

I am very glad, indeed, that the essayist has brought this kind of a subject before us, because it shows that an effort is being made to solve this problem on lines which will eventually relieve us from the danger of making very serious mistakes in the use of these substances. It is a serious mistake to have these accidents with so-called porcelain materials, because then all we hear is "Porcelain is no good;" "Porcelain has been a failure in my hands," etc., and you will find that the man has not used it intelligently or that he has used an improper material, and he therefore condemns the whole process. If we can know scientifically and practically what we should concerning porcelain, it will take the place it ought to make for itself.

*Dr. Hickman.*—I have enjoyed this paper, and I have learned a great deal from it. I came here, however, to be converted, and I am still among the lost. I cannot understand why it is that these other Dresden disciples do not speak, because the room is full of them. It does seem to me a strange thing that these failures of the low-fusing porcelain, the decompositions of the low-fusing porcelain, and these accidents on account of which porcelain is condemned, all come through the men who use the high-fusing porcelain. You do not often hear of the man who is using the low-fusing porcelain speak of this continual disintegration.

The essayist speaks of the careless manner in which we use the low-fusing porcelain as being one of the principal things against it. That is just the trouble. It is more difficult to make a good low-fusing porcelain inlay than to make a high-fusing one. Dr. Head is a high-fusing porcelain worker and he has had several men at conventions as exhibitors of the work, and at any angle you can see particles of food between the teeth. There is a gentleman in the room, who is much too modest to speak, who has put in at least a dozen porcelain inlays in one mouth, and at two feet from that patient I will defy any one to tell that the teeth have been filled. That is done with Jenkins low-fusing porcelain. He has done the most beautiful porcelain work in Philadelphia, but he does it with low-fusing porcelain and a gas-furnace. I do not use a gas-furnace entirely, especially when I bake porcelain on crowns. I use the Jenkins porcelain and an electric furnace. When you heat the whole body there is not the danger of fracture spoken of by the essayist, and I have yet to see a fracture of that kind. Only a short time ago while using a tooth made by the Consolidated Dental Manufacturing Company, in baking some Jenkins porcelain upon it, the body of the tooth split. Part of the enamel of the tooth broke off and was left on the porcelain instead of splitting off at the joint. I frequently use the Jenkins body in fitting a porcelain crown to a root, and I have yet to see an accident of that kind.

*Dr. Gaylord.*—I know from what Dr. Hickman said that the failures of low-fusing porcelain come from the men who are advocates of high-fusing porcelain, and Dr. Wheeler is going to tell him that the reason they are using the high-fusing is because they failed with the low. I do not think that is so. I cannot be a close-communionist, or a hard-shell, but am more of a universalist and so I use both high- and low-fusing. I feel that both have a place in dentistry, that failures are bound to exist in the use of both, but that satisfactory and good results can be produced with either.

I do not think that all of the arguments against low-fusing bodies will stand muster. I have yet to see Jenkins porcelain or any other low-fusing porcelain disintegrate in the mouth. I have had the pleasure of doing work for a family who some five years ago had porcelain work done by Dr. Jenkins himself, and the fillings have certainly stood five years' wear in the most beautiful manner. The surface of the porcelain is as perfect as any one could wish it to be. The lustre is good, and there is not a sign of disintegration. I do not mean to say that disintegration will not occur with low-fusing body, but I have yet to see it. If low-fusing porcelain is properly used it will turn out a good filling-material and one which will last. It is not so much what we use as how we use it. I could not well get on without using porcelain in my office. I am not an enthusiast, but when the opportunity presents itself I put porcelain in; sometimes the high-fusing, sometimes the low-fusing.

I read a little article in a Western dental journal in regard to the swaging of the matrix, which I have tried and found very helpful. The idea is to first make a pellet of cotton and carry the matrix to the bottom of the cavity, then to withdraw the cotton and finish the swaging with gum camphor. It is an ideal material to swage out the matrix. Fill the cavity nearly full before paying much attention to the margins. After the edges are worked down, finally fill the cavity with camphor. Your matrix has a stable material which helps to retain its form while removing it. The camphor burns out, leaving no ash, and the matrix is perfectly clean.

*Dr. Wheeler* (closing).—Dr. Gaylord very kindly made my answer on one point. I will continue what he had in mind. I have not said that low-fusing porcelain would not serve the purpose for which it was used in any or all cases, but I do know that the first case of disintegration in Jenkins porcelain I ever saw was put in by Dr. Jenkins himself at his office in Dresden. I know a young man in New York who has one of the most desirable practices in America, and he says that Jenkins enamel sometimes disintegrates in his hands and turns black. It does not go to pieces, but it loses any claim to being æsthetic that it may have had when put in. I have sometimes thought that its tendency to be opaque might be an advantage in certain cases, because backing it with opaque cement does not change its shade so badly as high-fusing material.

The question is, there are certain principles that I am sure are true, for instance, phenol sodique, if put into a bottle with a glass stopper, will make it difficult to remove the stopper. This shows you what a strongly alkaline fluid will do against glass. Dr. Kirk says it is hard to tell where glass ends and porcelain begins. I have given a definition something like this, which, to my mind, in a general way describes the situation as clearly as anything I have ever seen. I contend that anything in which all the ingredients fuse in the baking is a glass; but, if proportionate parts of that fusible material are not fused, and remain, as it were, as a sort of matrix or framework, that is a porcelain. It is possible, you must remember, to take silica, and by adding a sufficient quantity of basic alkaline salt, to dissolve the whole thing and make it into glass, because, so far as I know, the compounds are soluble in alkaline solutions.

In the matter of shrinkage, I have this afternoon learned from Dr. Guilford that in some experiments he had made, after a certain temperature there is no more shrinkage. This was never distinctly settled to my mind before. I have often wondered if my first bakes, which I never carry to a thoroughly smooth glaze, would shrink. I find that when you get to the point where it is baked, even though not glazed, the shrinkage ends; that is, in a true porcelain. Of course, you get a great change of shape with a glass. Drs. Hickman and Gaylord spoke about the high-fusing people being the ones who found fault with, or knew of cases of disintegration of low-fusing enamel. I hardly think that is so. I think any one who has kept a careful record of his work with low-fusing material will find cases in which there are marks of disintegration taking place. It is like cement. There may be mouths in which it never occurs, but again there are mouths in which it will occur. The line between high- and low-fusing bodies is an indistinct one, and there are a great many conditions that have a bearing on the question of the length of service of a porcelain inlay. The lower the point at which you get the glaze, the greater the probability that you are going to have the surface affected. All my experiments will bear me out in this.

Otto E. Inglis,
*Editor Academy of Stomatology.*

